# Two strategies by epiphytic orchids for maintaining water balance: thick cuticles in leaves and water storage in pseudobulbs

**DOI:** 10.1093/aobpla/plw046

**Published:** 2016-08-02

**Authors:** Shi-Jian Yang, Mei Sun, Qiu-Yun Yang, Ren-Yi Ma, Jiao-Lin Zhang, Shi-Bao Zhang

**Affiliations:** 1 Key Laboratory of Economic Plants and Biotechnology, Kunming Institute of Botany, Chinese Academy of Sciences, Kunming, Yunnan 650201, China; 2 Key Laboratory of Tropical Forest Ecology, Xishuangbanna Tropical Botanical Garden, Chinese Academy of Sciences, Mengla, Yunnan 666303, China; 3 National Plateau Wetlands Research Centre, Kunming, Yunnan 650224, China

**Keywords:** Cuticles, Dendrobium, pseudobulb, relative water content, water conservation, water loss

## Abstract

Epiphytes are an important component of tropical and subtropical flora, and serve vital ecological functions in forest hydrology and nutrient fluxes. However, they often encounter water deficits because there is no direct contact between their roots and the soil. The strategies employed by epiphytes for maintaining water balance in relatively water-limited habitats are not completely understood. In the present study, we investigated the anatomical traits, water loss rates, and physiology of leaves and pseudobulbs of four *Dendrobium* species with different pseudobulb morphologies to understand the roles of leaf and pseudobulb in maintaining water balance of epiphytic orchids. Our results showed that two species (*D. chrysotoxum* and *D. officinale*), with lower rates of water loss, have thicker leaves and upper cuticles, but lower epidermal thickness and leaf dry mass per area. In contrast, the other two species (*D. chrysanthum* and *D. crystallinum*) with thinner cuticles and higher rates of water loss, have less tissue density and greater saturated water contents in their pseudobulbs. Therefore, our results indicate that these latter two species may resist drought by storing water in the pseudobulbs to compensate for their thin cuticles and rapid water loss through the leaves. Under the same laboratory conditions, excised pseudobulbs with attached leaves had lower rates of water loss when compared with samples comprising only excised leaves. This implies that epiphytic orchids utilize two different strategies for sustaining water balance: thick cuticles to conserve water in leaves and water storage in pseudobulbs. Our results also show that *Dendrobium* species with thin cuticles tend to have pseudobulbs with high water storage capacity that compensates for their faster rates of water loss. These outcomes contribute to our understanding of the adaptive water-use strategies in *Dendrobium* species, which is beneficial for the conservation and cultivation of epiphytic orchids.

## Abstract (precis)

Epiphytes often encounter water deficits because there is no direct contact between their roots and the soil. However, it is still not fully understood how epiphytes maintain their water balances. In the present study, we found two different strategies for sustaining water balance employed by epiphytic orchids: thick cuticles to conserve water in leaves and water storage in pseudobulbs. The species with thin cuticles tend to have pseudobulbs with high water storage capacity that compensates for their faster rates of water loss. These outcomes are beneficial for the understanding of the adaptive water-use strategies in epiphytes.

## Introduction

Vascular epiphytes live non-parasitically on other living plants for support, and are often rich in tropical forests (Benzing 1990). They play critical roles in the hydrological and nutrient cycling of forest ecosystems. The biomass produced by vascular epiphytes in many canopies can be significant, especially in tropical montane cloud forests (Nadkarni *et al.* 2004; Gotsch *et al.* 2015). As structural elements of forests, these epiphytes are important for maintaining biological diversity and abundance because they provide food and habitats for numerous birds, insects and mammals in the forest canopy (Nadkarni and Matelson 1989; Sillett 1994; Treseder *et al.* 1995; Ellwood *et al.* 2002).

Many vascular epiphytes found in tropical and subtropical regions normally grow on tree trunks in forests and/or on rock surfaces in valleys (Benzing 1990). Unlike terrestrial plants, their roots have no contact with the soil. Therefore, water stress is a major limitation to their survival, growth and distribution (Zotz and Hietz 2001; Laube and Zotz 2003; Zotz and Bader 2009). However, vascular epiphytes exhibit more drought tolerance characteristics than do terrestrial species (Rada and Jaimez 1992; Watkins and Cardelús 2012; Zhang *et al.* 2015), even though the water-adaptive strategies may differ among epiphytic species.

For adapting to limited water availability, vascular epiphytes utilize suites of morphological and anatomical adaptations in their leaves, stems and roots, including thickened cuticles, stomata surrounded by trichomes, a reduction in transpiring surfaces through symodial growth, and development of aerial root systems (Moreira *et al.* 2013). The leaf cuticles of vascular epiphytes may act as efficient barriers against water loss after their stomata close (Helbsing *et al.* 2000). Furthermore, these types of epiphytes have greater capacity for foliar water uptake and tend to have thinner hydrenchymal and cuticle layers (Gotsch *et al.* 2015). Other mechanisms for storing water might involve the production of tissues such as succulent leaves and pseudobulbs, which are common features in vascular epiphytes (Sinclair 1984; Zimmerman 1990; Stern and Morris 1992). The hydrenchyma in succulent leaves from numerous epiphytes can store water to buffer the effects of reduced water supplies during periods of drought (Zotz and Bader 2009; Ogburn and Edwards 2010).

In addition to these multiple morphological and anatomical adaptations to water shortage, vascular epiphytes demonstrate differences in their phenology and photosynthetic pathways. In Orchidaceae, some deciduous epiphytic orchids may react to relatively long-lasting drought by shedding their leaves to reduce the surface area available for transpiration. Diurnal water losses from stems of plants without leaves are only ∼30 % of the amount measured from stems of plants with leaves, such as occurs with the epiphytic orchid *Dimerandra emarginata* (Zotz and Tyree 1996). When compared with the performance of C_3_ photosynthesis, crassulacean acid metabolism (CAM) in many epiphytes plays an important role in improving carbon gains and water use (Motomura *et al.* 2008*a*). Furthermore, the CAM pathway of photosynthesis can enable plants to harvest external CO_2_ at low cost of water (Reinert 1998; Lüttge 2004).

In some orchids, pseudobulbs are very short, thick and bulb-shaped stems that can bear one or more leaves at the top or along the stem axes. These structures are present among most of the epiphytic orchids in tropical and subtropical regions. As storage organs for water, carbohydrates and minerals, pseudobulbs are of central importance to the growth and survival of those orchids (Goh and Kluge 1989; Ng and Hew 2000). Furthermore, they have critical roles during the processes of vegetative growth, flowering and reproduction (Zimmerman 1990; Blanchard and Runkle 2008). For trees, internal water stored in the stems contributes significantly to regulating their daily transpirational water losses (Goldstein *et al.* 1998). Likewise, water stored in the pseudobulbs of the epiphytic orchids may constitute an effective reservoir for protecting those plants against the effects of short- or long-term water deficits. Despite the recognized value of pseudobulbs, however, most published studies on the water relations of vascular epiphytes have historically focused primarily on their leaves (e.g. Sun *et al.* 2014; Zhang *et al.* 2015) and few researchers have examined how pseudobulbs help maintain water balance.

The Orchidaceae is the largest and most varied families of epiphytic vascular plants, particularly because of their life forms, habitats, and morphological and physiological traits (Benzing 1990). Approximately 68 % of all vascular epiphytes are orchids and they are widely distributed in tropical and subtropical regions (Zotz 2013). The genus *Dendrobium*, one of the largest genera within the Orchidaceae, comprises ∼1000 species, with 78 species occurring in China (Ji 1999). Most members of *Dendrobium* are naturally distributed throughout South-, East- and Southeast Asia, as well as the Southwest Pacific islands. *S*pecies within that genus exhibit a sympodial growth habit, with thickened organs (succulent leaves and/or pseudobulbs) that can store enough moisture so that plants can survive for several months without rainfall. In this study, we selected four epiphytic orchids (*Dendrobium* species) with different pseudobulb morphologies that were grown under the same environmental conditions in a nursery. Our main objectives were to: (1) quantify the role of pseudobulbs in buffering water losses from the leaves and (2) characterize the structural and physiological traits used by these epiphytic orchids to conserve water and maintain water balance. The goal of our investigation was to obtain new information that could expand our understanding of adaptive water-use strategies by epiphytic orchids.

## Methods

### Plant materials and experimental conditions

**Figure 1.**
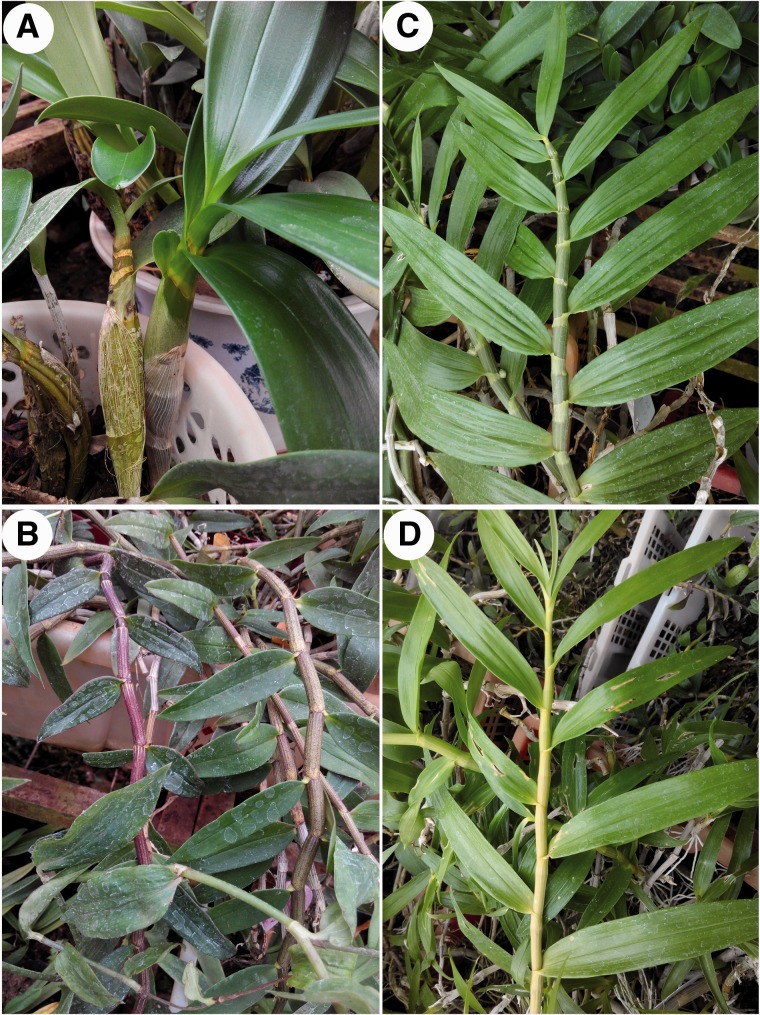
Leaves and pseudobulbs of four studied *Dendrobium* species: (A) *D. chrysotoxum*, (B) *D. officinale*, (C) *D. chrysanthum* and (D) *D. crystallinum*.

This study was carried out at the Kunming Institute of Botany (KIB), Chinese Academy of Sciences (25°01′N, 102°41′E, 1900 m above sea level), located at Kunming City, Yunnan Province, in southwestern China. All plant materials of the four *Dendrobium* species (*D. chrysanthum*, *D. chrysotoxum*, *D. crystallinum* and *D. officinale*) (Fig. 1) were collected from the nursery of Xishuangbanna Tropical Botanical Garden (21°41′N, 101°25′E) in southwestern China. These species were chosen because they are very common epiphytic orchids with different pseudobulb morphologies, such as a drum hammer shape for *D. chrysotoxum* or a slender form, as in *D. crystallinum* (Fig. 1). According to the photosynthetic pathway classification by Pérez-Harguindeguy *et al.* (2013), the δ^13^C values of the four tested species indicated that the photosynthetic modes of *D. chrysanthum*, *D. chrysotoxum* and *D. crystallinum* are C_3_ patterns (δ^13^C values ranged from −21.0‰ to −35.0‰), while *D. officinale* (–20.9‰) shows facultative CAM photosynthetic pathway (Fig. 5C, the details were given in the Discussion section). Our study plants were placed in perforated plastic pots filled with dry coconut shells, tree bark and humus as substrates and were maintained in a KIB orchid greenhouse at a mean temperature of 25 °C, relative humidity of 75 % and photosynthetically active radiation of 400 μmol m ^−^ ^2^ s ^−^ ^1^ in the daytime. The plants were watered at least once a week, and fertilized monthly through the leaves with common nutrient solutions. After growing within this greenhouse setting for >1 year, it was assumed that the sampled plants were fully acclimated to these environmental conditions by the time the experiments began.

**Figure 2.**
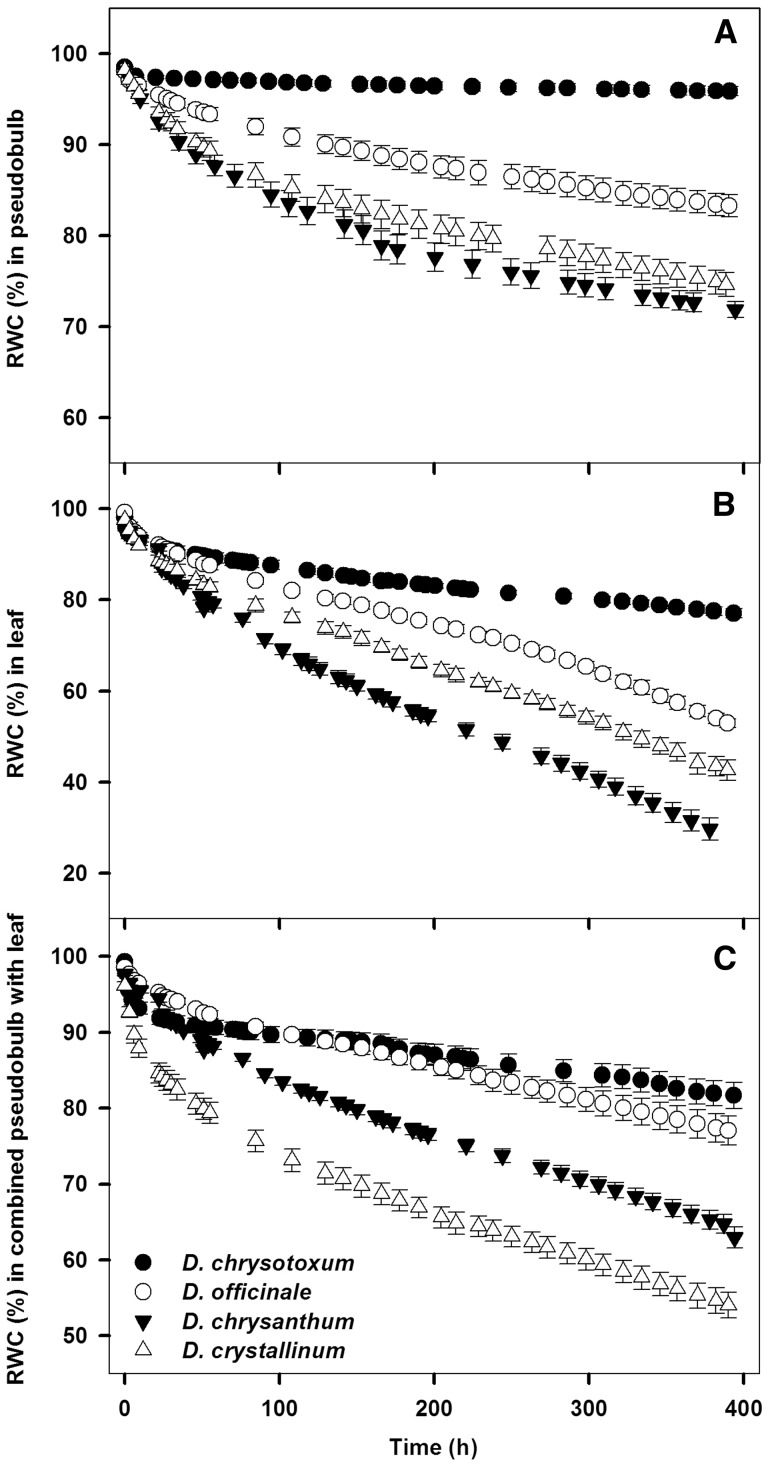
Water loss curves for leafless pseudobulb, excised leaf only and pseudobulb with attached leaves from four *Dendrobium* species (D. *chrysotoxum*, D. *officinale*, D. *chrysanthum* and D. *crystallinum*), showing changes in relative water content (RWC) with time (h) after excision.

### Water loss curves and relative water content of pseudobulbs and leaves

The rates of water loss after excision were measured for pseudobulbs with and without leaves as well as for detached mature leaves. Pseudobulbs with their leaves still attached were excised in late afternoon, sealed in zip lock bags and immediately transported to the laboratory for rehydration overnight with distilled water. Following rehydration, the samples were treated in three ways: pseudobulbs with leaves attached, pseudobulbs with leaves removed and only the excised leaves themselves. After the severed ends of the pseudobulbs and detached leaves were sealed with Parafilm, fresh weights at saturation (FW_s_) were measured for all samples. The tissues were then placed on a laboratory bench. For determining rates of water loss, fresh weights (FW) were obtained periodically on a digital balance, and the time of measurement was also recorded. At the end of this monitoring period, the area of each leaf was assessed with a Li-3000A Portable Area Meter (Li-Cor, NE), and all samples were then oven-dried at 80 °C for 48 h before their dry weights (DW) were recorded. The relative water content (RWC) was calculated as (FW−DW)/(FW_s_−DW) × 100 %. Water-loss curves were obtained by plotting changes in RWC against time.

### Leaf anatomical traits and calculations of leaf dry mass per unit area

Mature leaves from six individuals were fixed in FAA (formaldehyde: glacial acetic acid: alcohol = 5:5:90). Transverse cross sections were made by cutting vertically the middle part of the leaf with a sharp razor blade. The transverse sections were stained in a 0.05 % toluidine blue solution for at least 2 min, and then transferred to microscope slides, rinsed with pure water and photographed at 200× and 400× magnifications with a light microscope (Leica DM2500, Germany). Digital photos were scaled and analyzed with Image J software (http://rsbweb.nih.gov/ij/) to determine leaf thickness (LT), upper cuticle thickness (UCT), upper epidermal thickness (UET) and lower epidermal thickness (LET).

For characterizing stomatal traits, the abaxial, middle portions of the leaves were pasted onto pellucid enamels, and then transferred to microscope slides after drying. The stomatal prints on the enamels were captured at 200× and 400× magnifications under a light microscope (Leica DM2500, Germany). From 30 randomly selected digital images for each species, stomatal length (SL; namely, length of the guard cells) was obtained with aforementioned software, and stomatal density (SD) was calculated as the number of stomata per unit leaf area.

After the enamels for measuring the stomata were removed completely, the same leaves were used to examine vein density (VD). The mesophyll tissues were removed by boiling the middle segments (∼3 cm^2^) of the leaves for 20 min in a 5 % NaOH aqueous solution and then soaking them for 30 min in distilled water. After bleaching for 20 min in 5 % sodium hypochlorite, they were again soaked for 30 min in distilled water. The prepared samples were dyed with 1 % toluidine blue for 2 min, transferred to slides and photographed at 100 × magnification under the Leica DM2500 light microscope. Values for VD were calculated as the total length of leaf vascular tissue per leaf area after the total vein length was determined from digital images with aforementioned software.

Leaf areas (cm^2^) were also measured from other mature leaves with the Li-3000A Portable Area Meter. These samples were then oven-dried at 80 °C for 48 h to a constant dry mass. The leaf dry mass per unit area (LMA; g m ^−^ ^2^) was calculated as the ratio of leaf dry mass to leaf area.

### Leaf gas exchange, water use efficiency and stable carbon isotope composition

The leaf maximum net photosynthetic rate (A), stomatal conductance (g_s_) and transpiration rate (E) were monitored from six selected individuals per species, using a Li-6400 portable photosynthetic system (LI-COR Biosciences Inc., Lincoln, NE). The stomatal aperture was maximized by exposing the mature leaves to a light intensity of 300 μmol m ^−^ ^2^ s ^−^ ^1^ irradiance for 30 min. All measurements were made between 09:30 and 11:30 h on clear days. During the measurement periods, steady photosynthetic photo flux density was set at 300 μmol m ^−^ ^2^ s ^−^ ^1^, based on light saturation points from light response curves for these *Dendrobium* species. The temperature and CO_2_ concentration in the leaf chamber were maintained at 25 °C and 380 μmol mol ^−^ ^1^, respectively. The intrinsic water use efficiency (WUE_i_) was calculated as the ratio of A to E.

To investigate the photosynthetic pathway and estimate long-term water use efficiency of the leaves, mature, undamaged leaves from all four *Dendrobium* species were collected, oven-dried at 80 °C and then pulverized to a fine powder through a sieve with holes of 0.25 mm in diameter. The leaf stable carbon isotope composition (δ^13^C) was analyzed with an isotope mass spectrometer (Isoprime 100; Cheadle Hulme, UK).

### Saturated water content and tissue density of pseudobulbs and leaves

Water storage capacity was quantified by calculating the saturated water content (SWC) of samples (pseudobulbs and/or leaves) as (FW_s_−DW)/DW, where FW_s_ is the fresh weight of the tissue at saturation and DW is the constant dry mass.

Pseudobulb tissue density was measured from five or six individuals per species. At predawn, pseudobulbs with leaves were taken from each species, sealed in plastic bags and were immediately transported to the laboratory. After the leaves were removed with scissors, each pseudobulb was placed in water in a graduated cylinder to determine its volume, then oven-dried to a constant mass and weighed to obtain the dry mass. The pseudobulb tissue density (g cm ^−^ ^3^) was determined by dividing the dry mass by the volume of the sampled pseudobulbs. Leaf tissue density (g cm ^−^ ^3^) was calculated as LMA/LT, where LMA (g m ^−^ ^2^) is leaf mass per unit area, and LT (cm) is leaf thickness.

### Statistical analyses

The results were presented as mean values for five or six individuals from the each of four sampled *Dendrobium* species. Differences among species for RWC, anatomical traits, stomatal characters, gas exchange parameters, WUE_i,_ δ^13^C values, saturated water content and tissue density of leaves and pseudobulbs were determined by one-way analysis of variance (ANOVA) and LSD multiple comparisons tests in SPSS 16.0 statistical software (SPSS Inc., Chicago, IL).

## Results

### Rates of water loss and relative water content of pseudobulbs and leaves

Under the same laboratory conditions, the rates of water loss from the excised leafless pseudobulbs and the detached leaves differed among the four *Dendrobium* species, following the order (from slow to fast) of *D. chrysotoxum *<* D. officinale *<* D. crystallinum *<* D. chrysanthum*. However, when the leaves remained attached to the pseudobulbs, changes in relative water content (RWC) over time were similar between *D. chrysotoxum* and *D. officinale*, whereas the water losses were considerably faster for *D. crystallinum* than for *D. chrysanthum* (Fig. 2).

**Figure 3.**
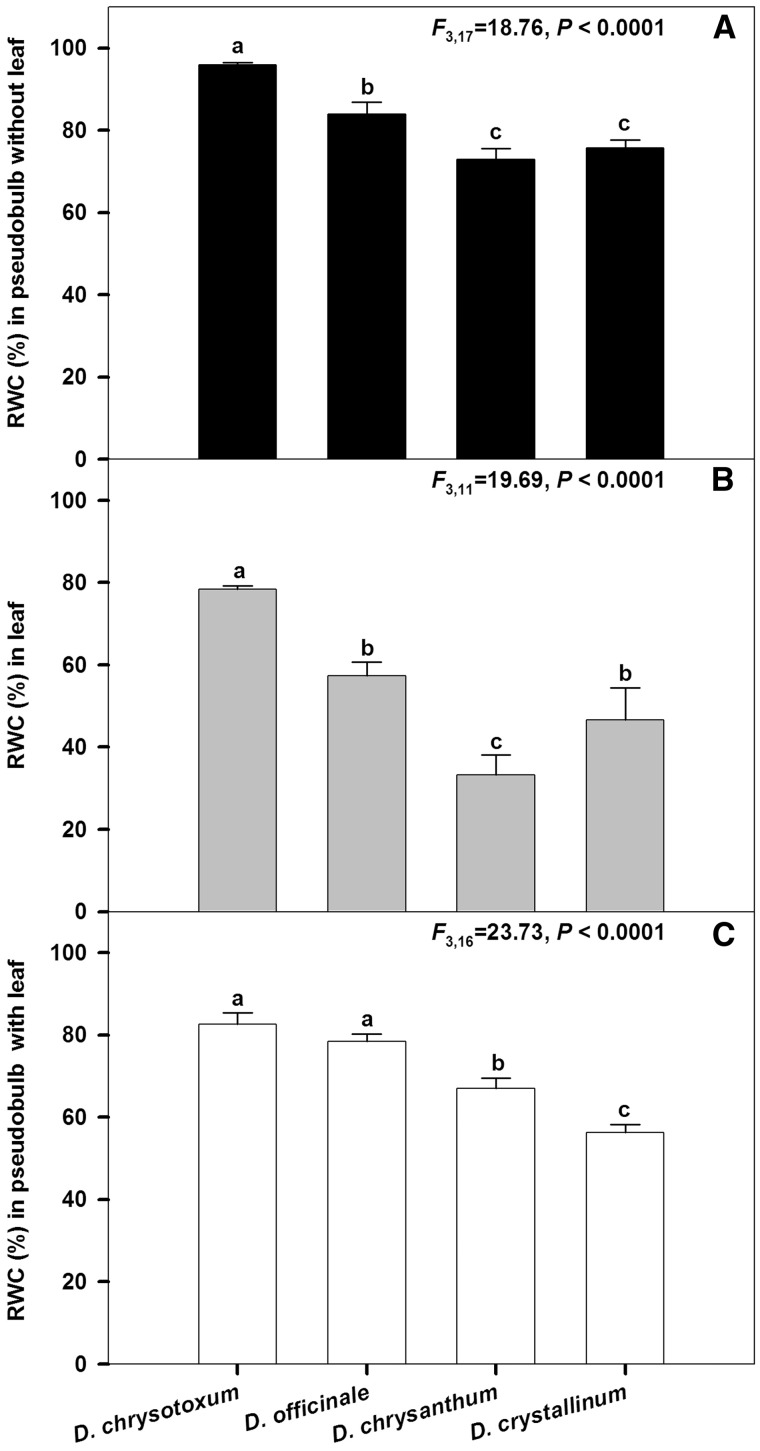
Relative water content (RWC, %) in leafless pseudobulb, excised leaf only and pseudobulb with attached leaves from four *Dendrobium* species (D. *chrysotoxum*, D. *officinale*, D. *chrysanthum* and D. *crystallinum*) at 15 days after organs were excised under the same laboratory conditions. Bars are means±SE. For each sample type, significant differences (P<0.0001) among four species are indicated by different letters.

When pseudobulbs without or with leaves of four *Dendrobium* species were excised and dehydrated after 15 days in the laboratory, their RWC values were obviously different (*P *< 0.0001; Fig. 3). For *D. chrysotoxum*, the leafless pseudobulbs as well as the excised leaf samples still retained their relatively high RWC values (95.9 % and 78.4 %, respectively). However, for *D. chrysanthum*, the RWC of those excised organs dropped to 72.9 % and 33.2 %, respectively (Fig. 3).

**Figure 4.**
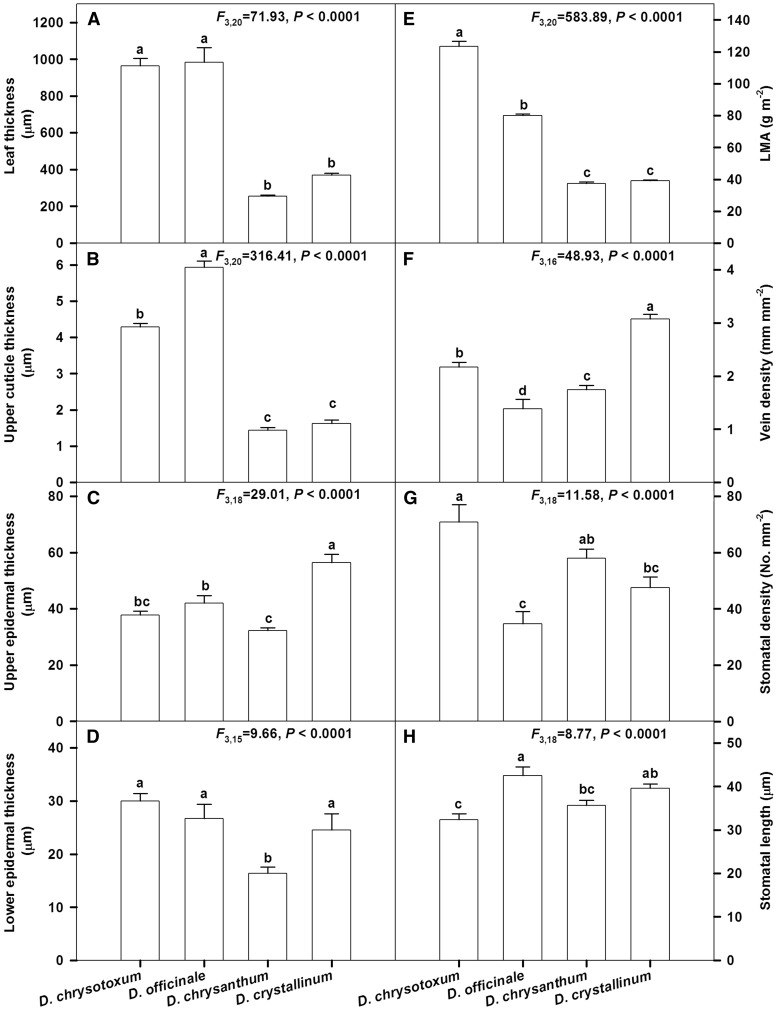
Leaf anatomical traits, stomatal characters and leaf dry mass per unit area (LMA) of four *Dendrobium* species (*D. chrysotoxum*, *D. officinale*, *D. chrysanthum* and *D. crystallinum*). Data are mean±SE. Significant differences (P<0.0001) among species are indicated by different letters.

### Leaf anatomical traits and LMA

Among the four *Dendrobium* species, the leaves of *D. chrysotoxum* and *D. officinale* had comparatively higher values for LT, UCT, LET and LMA (*P *< 0.0001; Fig. 4A, B, D and E). Compared with the other three species, values were significantly higher for UET (*F*_3,18 _=_ _29.01, *P *< 0.0001; Fig. 4C) and VD (*F*_3,16 _=_ _48.93, *P *< 0.0001; Fig. 4F) of *D. crystallinum*. The SD ranged from 34.75 No. mm ^−^ ^2^ in *D. officinale* to 70.83 No. mm ^−^ ^2^ in *D. chrysotoxum*, while SL ranged from 32.31 μm in *D. chrysotoxum* to 42.52 μm in *D. officinale* (Fig. 4G and H).

**Figure 5.**
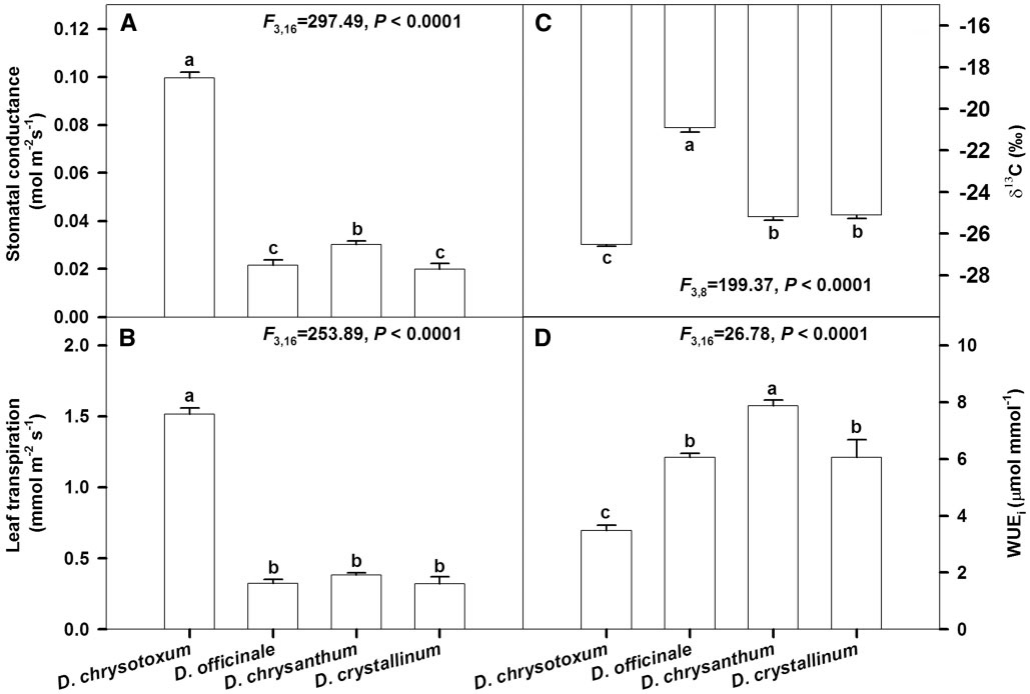
Leaf maximum stomatal conductance, transpiration rate, stable carbon isotope composition (δ^13^C) and intrinsic water use efficiency (WUEi) of four *Dendrobium* species (*D. chrysotoxum*, *D. officinale*, *D. chrysanthum* and *D. crystallinum*). Data are mean±SE. Significant differences (P<0.0001) among species are indicated by different letters.

### Leaf gas exchange, water use efficiency and stable carbon isotope composition

In the sampled *Dendrobium* species, *D. chrysotoxum* showed the highest leaf maximum stomatal conductance (*F*_3,16 _=_ _297.49, *P *< 0.0001; Fig. 5A) and transpiration rate (*F*_3,16 _=_ _253.89, *P *< 0.0001; Fig. 5B). The δ^13^C values of *D. chrysanthum*, *D. chrysotoxum*, *D. crystallinum* and *D. officinale* were −25.2‰, −26.5‰, −25.1‰ and −20.9‰, respectively (Fig. 5C). The intrinsic water use efficiency (WUE_i_) was highest for *D. chrysanthum* and lowest for *D. chrysotoxum*, while WUE_i_ values were in the mid-range for both *D. officinale* and *D. crystallinum* (*F*_3,16 _=_ _26.78, *P *< 0.0001; Fig. 5D).

### Saturated water content and tissue density of pseudobulbs and leaves

**Figure 6.**
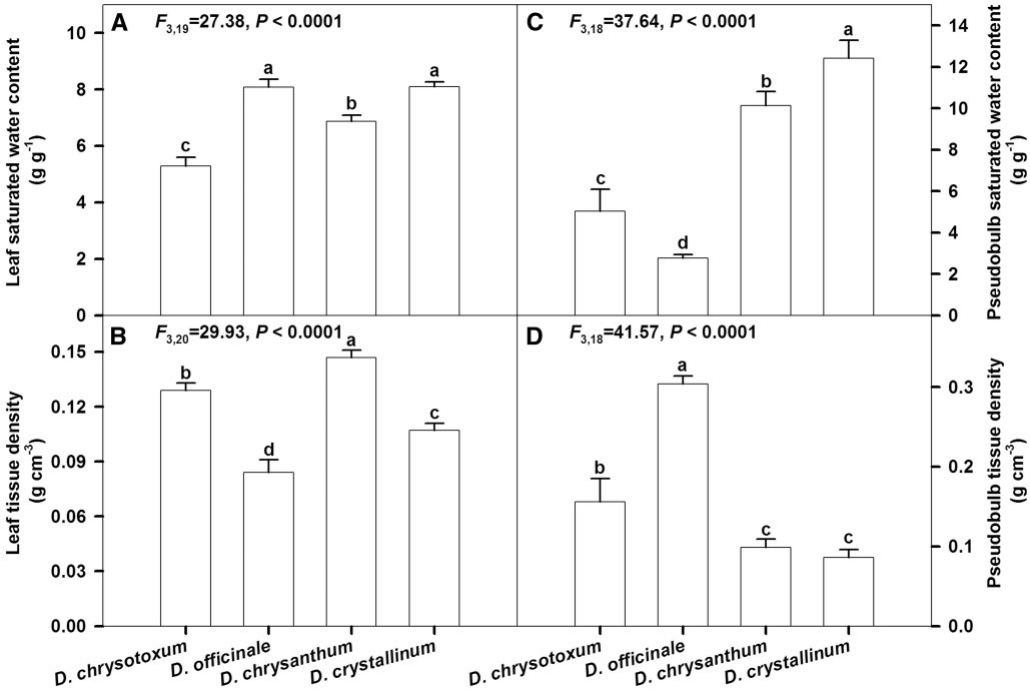
Saturated water content and tissue density of leaf (A, B) and pseudobulb (C, D) of four *Dendrobium* species (*D. chrysotoxum*, *D. officinale*, *D. chrysanthum* and *D. crystallinum*). Data are mean ± SE. Significant differences (*P *< 0.0001) among species are indicated by different letters.

Leaf SWC and tissues density were lowest for *D. chrysotoxum* (*F*_3,19 _=_ _27.38, *P *< 0.0001; Fig. 6A) and *D. officinale* (*F*_3,20 _=_ _29.93, *P *< 0.0001; Fig. 6B), respectively. For pseudobulbs, interspecific differences were significant in SWC (*F*_3,18 _=_ _37.64, *P *< 0.0001), with the highest value (12.42 g g ^−^ ^1^) in *D. crystallinum* and the lowest value (2.76 g g ^−^ ^1^) in *D. officinale* (Fig. 6C). Similar to the species-specific differences in leaf tissue density, pseudobulb tissue density of sampled *Dendrobium* species varied widely, from 0.086 g cm ^−^ ^3^ in *D. crystallinum* to 0.304 g cm ^−^ ^3^ in *D. officinale* (*F*_3,18 _=_ _41.57, *P *< 0.0001; Fig. 6D).

## Discussion

Epiphytic orchids tolerate temporary water deficits and prevent excessive transpirational losses by adapting both morphologically and metabolically. Although all of our tested *Dendrobium* species are epiphytes with pseudobulbs, their strategies for maintaining water balance differ significantly. Our results showed that the thick leaf cuticles in *D. chrysotoxum* and *D. officinale* play important roles in protecting against water losses through evapotranspiration. Consequently, these two species have the lowest rates of water loss. In contrast, the other two species (*D. chrysanthum* and *D. crystallinum*) with thin cuticles and high water loss rate, unilize pseudobulbs with high water-storing capacity to maintain water balance. Our study suggests that different mechanisms are employed by different epiphytic orchid species to cope with habitats where water supplies are limited.

### The function of thick cuticles as a barrier of resistance to water evaporation

Leaf cuticles seem to have an important role for the reduction in relative water content. In epiphytes, cuticle thickness is related to a plant’s capacity for foliar water uptake (Gotsch *et al.* 2015). As an early plant response to water deficits, low stomatal conductance means that the transpiration rate is reduced. Thick leaf cuticle is the main characteristic responsible for preventing water losses after stomatal closure. In some *Dendrobium* species, deploy thick, evaporation-reducing cuticles covered the adaxial epidermis serve as a first line of defense against desiccation (Sun *et al.* 2014). It suggests that the epiphytic orchids with thicker cuticles lose water very slowly when they encounter water stress. In addition, a higher LMA and thicker leaves help in conserving water by decreasing the area of leaf surface that is susceptible to evapotranspiration.

In a recent study, it was found that the time required for drying saturated leaves to 70 % RWC in epiphytic *Cymbidium* species was longer than that in terrestrial species (Zhang *et al.* 2015). Furthermore, it was also reported that the RWC for two epiphytic orchids (*Dendrobium tortile* and *D. crumenatum*) still maintain >90 % even after 20 days of drought (Sinclair 1983*b*). In the leaves and pseudobulbs of another epiphytic orchid (*Cattleya forbesii*), a decrease in RWC and a concomitant increase in dry mass/fresh mass ratio was observed under conditions of water deficit (Stancato *et al.* 2001). It has been demonstrated that the thickness of the cuticle is correlated with cuticular water permeability and the extent to which a plant can avoid water losses (Kerstiens 1996; Riederer and Schreiber 2001). We also found that the thicker leaves and cuticles of *D. chrysotoxum* and *D. officinale* (Fig. 4A and B) confer more efficient protection against the loss of water. Under water stress, the RWC of leaves and pseudobulbs are decreased significantly, but they are still maintained at a relatively high level. This is probably associated with the low cuticular permeability to water that most epiphytic orchids exhibit (Helbsing *et al.* 2000).

### The function of pseudobulbs as a water reserve to maintain water balance

Compared with samples of individual excised leaves, the pseudobulbs that still had attached leaves had higher RWC values during our desiccation treatment (Fig. 3). This indicated that water stored in the pseudobulbs is transported to the leaves under such stress conditions. Thus, we might conclude that these pseudobulbs release stored water to meet the transpirational demands of the leaves. The water held in those pseudobulbs of epiphytic orchids then constitutes an effective reservoir for buffering plants against the effects of drought. Pseudobulbs may function to slowly decrease leaf water content and water potential under conditions of water deficit (Goh and Kluge 1989; Zheng *et al.* 1992). Furthermore, the abundant parenchyma cells in those pseudobulbs are another part of a mechanism that epiphytic orchids use to adjust to water deficit (Pires *et al.* 2012). Our findings are consistent with those previously reported that the pseudobulbs facilitate a slow reduction in leaf water content of *Cymbidium sinense* during a period of drought (Zheng *et al.* 1992).

By comparison, species with thin cuticles (*D. chrysanthum* and *D. crystallinum)* have pseudobulbs with lower tissue density and higher saturated water content (Fig. 6). Those traits allow plants that lose water relatively fast to adapt to epiphytic environments with probable higher water deficits. The saturated water content of tissues is considered an indicator of water storage capacity (Stratton *et al.* 2000). Here, higher saturated water content in the pseudobulbs of *D. chrysanthum* and *D. crystallinum* meant that more water was available to the plants. Pseudobulbs in epiphytic orchids not only serve in water maintenance, but are also associated with a reserve for carbohydrates. Under drought conditions, water–soluble polysaccharides in the pseudobulbs are mobilized so that plants can tolerate relatively long periods of stress (Stancato *et al.* 2001).

### Other mechanisms enable epiphytic orchids to adapt to water deficits

Leaf stomatal characteristics, such as stomatal density and length, are involved in stomatal conductance and transpiration. Compared with leaves from other angiosperms, the stomatal density is relatively low in members of Orchidaceae, including species of *Cypripedium*, *Paphiopedilum*, *Dendrobium* and *Cymbidium* (Guan *et al.* 2011; Zhang *et al.* 2012, 2015; Sun *et al.* 2014). Therefore, those plants can tolerate more severe water deficits than species with higher stomatal density. The relatively high stomatal conductance and transpiration rate in *D. chrysotoxum* probably result mostly from its relatively high stomatal density (Fig. 4G). Our four sampled epiphytic orchids also vary in their instantaneous water-use efficiencies (WUE_i_), perhaps because of differences in their leaf morphological characteristics. For *D. chrysotoxum*, this includes having the smallest stomata and the highest stomatal density (Fig. 4G and H). Smaller stomata close more rapidly, wick water more efficiently, and have greater potential for maintaining hydraulic functioning during drought periods (Aasamaa *et al.* 2001).

Compared with our other sampled epiphytic orchids, the stomatal conductance (*g_s_*) and leaf transpiration rate (E) in *D. chrysotoxum* is higher under relatively low light intensity (Fig. 5A and B). This indicates that the stomata of this orchid species are open during the day. Moreover, these findings are also consistent with δ^13^C values that are <−25‰ in this low WUE_i_ species(Fig. 5C and D). Altogether, these data provide evidence that the photosynthetic behaviour of *D. chrysotoxum* follows the pattern of a C_3_ plant. Compared with other C_3_ plant groups, the absolute values of *g_s_* and E are low in *Dendrobium* species, but are also similar to those observed in other epiphytic orchids (Pires *et al.* 2013).

Carbon isotope composition can be used as measures of photosynthetic pathway and intrinsic water-use efficiency (Silvera *et al.* 2009; Pérez-Harguindeguy *et al.* 2013). According to the photosynthetic pathway classification of Pérez-Harguindeguy *et al.* (2013), C_3_ photosynthesis species has a δ^13^C value ranging from −21‰ to −35‰, facultative CAM species from −15‰ to −20‰ and obligate CAM species from −10‰ to −15‰. Thus, the δ^13^C values suggested that the photosynthetic pathways of *D. chrysanthum*, *D. chrysotoxum* and *D. crystallinum* in the present study are likely C_3_ patterns, while *D. officinale* has concomitant CAM and C_3_ photosynthetic pathways. A previous study also suggested that the switch in CO_2_ assimilation patterns from C_3_ to CAM can be induced by drought stress (Zhang *et al.* 2014). Although the photosynthetic pathway of a species can be determined by C-isotope analysis, more methodological considerations such as anatomical observation and diurnal measurement of organic acid concentration would be also important (Pérez-Harguindeguy *et al.* 2013). The occurrence of water-conserving CAM in many epiphytic orchids improves water economy by stimulating the nocturnal opening of stomata (Motomura *et al.* 2008*a*). In the three other epiphytic CAM orchids (*Eria velutina*, *Dendrobium tortile* and *D. crumenatum*) studied by Sinclair, stomatal activity continues to maintained even under drought stress (Sinclair *1983a, b*). Although a CAM photosynthetic pathway is primarily associated with the leaf succulence of some species in arid habitats, it is not always correlated with a high leaf thickness in epiphytic orchids (Motomura *et al.* 2008*b*).

In addition to utilizing the water supplied from precipitation or dew, vascular epiphytes take up water from the atmosphere through their aerial roots (Zotz and Winkler 2013). We did not address this phenomenon specifically in our present study. Therefore, future explorations should focus on the velamen covered aerial roots of epiphytic orchids, which seems to be important for maintaining their water balance.

## Conclusions

In summary, our results reveal two strategies by *Dendrobium* species in maintaining water balance. Species with thick cuticles primarily use them as a barrier against water losses, while species with thin cuticles mainly use pseudobulbs for storing sufficient water to buffer rapid water losses from the leaves. Translocation of water between leaves and pseudobulbs may be an important mechanism for maintaining water balance in some epiphytic orchids during periods of drought. Our study results improve our understanding about the multiple mechanisms of water balance strategies in epiphytic orchids. Nevertheless, further research based on moisture controlled experiments is needed to investigate the important role of pseudobulbs in maintaining plant water balance. 

## Sources of Funding

Our research was financially supported by the National Natural Science Foundation of China (31170315, 31370362 and 31400340), the Natural Science Foundation of Yunnan Province (2013FA044), the China Postdoctoral Science Foundation Funded Projects (2014M560735 and 2015T80994) and the National Key Technology Research and Development Program of the Ministry of Science and Technology of China (2015BAD10B03).

## Contributions by the Authors

S.Y., M.S. and S.Z. designed the experiments. M.S. and Q.Y. performed the experiments. S.Y. and M.S. analyzed the data. S.Y., M.S., R.M., J.Z. and S.Z. contributed to the writing. All authors had intellectual input into the project.

## Conflict of Interest Statement

None declared.
